# Whole Genome Low-Coverage Sequencing Concurrently Detecting Copy Number Variations and Their Underlying Complex Chromosomal Rearrangements by Systematic Breakpoint Mapping in Intellectual Deficiency/Developmental Delay Patients

**DOI:** 10.3389/fgene.2020.00616

**Published:** 2020-07-06

**Authors:** Bing Xiao, Xiantao Ye, Lili Wang, Yanjie Fan, Xuefan Gu, Xing Ji, Yu Sun, Yongguo Yu

**Affiliations:** ^1^Department of Pediatric Endocrinology and Genetic Metabolism, School of Medicine, Shanghai Institute for Pediatric Research, Xinhua Hospital, Shanghai Jiao Tong University, Shanghai, China; ^2^Shanghai Key Laboratory of Pediatric Gastroenterology and Nutrition, Shanghai, China

**Keywords:** copy number variations, chromosome rearrangement, whole-genome low-coverage sequencing, intellectual disability, developmental delay

## Abstract

Simple copy number variations (CNVs) detected by chromosomal microarray (CMA) can result from complex structural changes. Therefore, it is necessary to characterize potential structural changes that cause pathogenic CNVs. We applied whole-genome low-coverage sequencing (WGLCS) to concurrently detect pathogenic CNVs and their associated chromosomal rearrangements in 15 patients. All the patients had an average of 2–3 pathogenic CNVs involving 1–2 chromosomes. WGLCS identified all the 34 pathogenic CNVs found by microarray. By identifying chimeric read pairs, WGLCS mapped 70 breakpoints in these patients, of which 47 were finely mapped at the nucleotide level and confirmed by subsequent PCR amplification and Sanger sequencing of the junction fragments. In 15 patients, structural rearrangements were defined at molecular level in 13 patients. In 13 patients, WGLCS reveal no additional results in two patients. In another 11 patients, WGLCS revealed new breakpoints or finely mapped the genes disrupted by breakpoints or 1–6 bp microhomology and/or short insertion (4–70 bp) in the breakpoints junctions. However, structural changes in the other two patients still remained unclear after WGLCS was performed. The structural alteration identified in the 13 patients could be divided into the following categories: (1) interstitial inverted duplication with concomitant terminal deletion (inv dup del) (P1,P4,P9,P11); (2) the product of pericentric inversion (P5); (3) ring chromosome (P8); (4) interstitial duplication and/or triplication (P6, P7); and (5) +der(22)t(11;22) (P2,P15); (6) complex structural rearrangements (P3,P12,P14). WGLCS displayed the ability to discover CNVs and define breakpoints and its disrupted genes and its surrounding sequences in one experiment at base-pair-resolution, which help us to learn more about the mechanisms of formation of observed genomic rearrangements, and in which DNA replicative/repair mechanism might contribute to the formation of complex rearrangements in 11 patients. Clear karyotype at molecular level could help provide an accurate evaluation of recurrent risk and guide prenatal diagnosis or reproductive planning.

## Introduction

Chromosomal microarray analysis (CMA) has been implemented as the first-tier diagnostic test for copy number changes in patients with intellectual disability/development delay (ID/DD), multiple congenital anomalies (MCA), and autism (Miller et al., 2010; Beaudet, 2013). With the introduction of microarray analysis in clinical diagnosis, large numbers of submicroscopic pathogenic copy number variants (CNVs) have been uncovered. However, in contrast to conventional karyotyping and fluorescence *in situ* hybridization (FISH), CMA cannot reveal the exact structural configuration of an abnormal chromosome, except for single deletions/duplications. Some pathogenic CNVs found by microarray may have underlying complex chromosomal rearrangements that are parental in origin, which can lead to high recurrent risk (Nowakowska et al., 2012). Therefore, it is necessary to elucidate the exact structural alterations presented in an individual personal genome.

In the molecular cytogenetics era, with the technological developments in genomics, next-generation sequencing (NGS) methods have allowed us to rapidly capture breakpoints which improve our understanding of the molecular basis of genomic disorder associated with structural variation (SV). In recent years, many reports on NGS-based methods for mapping breakpoints in ID/DD/MCA-associated chromosome rearrangements have been proposed and provides another choice in the characterization of complex chromosomal rearrangements at a high resolution (Chen et al., 2010; Sobreira et al., 2011; Schluth-Bolard et al., 2013; Vergult et al., 2014; Nilsson et al., 2017; Lindstrand et al., 2019). The whole-genome low-coverage sequencing (WGLCS) approach with large insert size (~4 kb) enables the detection of balanced chromosomal rearrangement events. It is independent of knowledge of the affected regions and could identify the breakpoints of complex chromosomal rearrangements at the nucleotide level (Li et al., 2014; Yang et al., 2014; Pan et al., 2016; Yao et al., 2016; Luo et al., 2018). In this study, we aim to systematically assess its ability in simultaneous detection of CNVs and their underlying complex chromosomal rearrangements at the single-base-pair level.

## Methods

### Patient Enrolment

The samples included in this study were all ID/DD patients who were referred to our lab for CMA test during Jan, 2014 to Dec, 2018. We selected patients with multiple pathogenic CNVs (*n* ≥ 2) involving 1-2 chromosomes detected by microarray which represent several CNV patterns. In total 15 patients were enrolled and retrospectively analyzed in this study. The study protocol was approved by the Ethics Committee of Xinhua Hospital, Shanghai Jiaotong University School of Medicine.

### Karyotype Analysis

Routine karyotype analyses of the patient and their parents were performed on GTG-banded (400–550 bands resolution) metaphases method from cultures of PHA-stimulated peripheral blood lymphocytes according to standard procedures. Karyotype analysis was only performed in five patients (P2, P8, P13, P14, and P15) and the results were listed in Table 1.

**Table 1:** Breakpoint junction features of the structural rearrangements identified in 15 patients.

**Patient number**	**Karyotype**	**CMA results (hg19)**	**Length (kb)**	**ACMG classification**	**Breakpoints number defined by WGLCS**	Verified by Sanger sequencing^*^	**Disrupted gene**	**Breakpoint characteristics**	**Structural rearrangements type**	**Final interpretation**
**(1) CASES WITH NEW RESULTS AFTER WGLCS**										
P1	ND	arr 13q31.2q34(88,892,801-112,091,733)x3	23,199	P	2	2	_	6 bp microhomology	Inv dup del	seq[GRCh37]+der(13)(pter → q34:: q34 → q31.2)
		arr 13q34(112,091,966-115,107,733)x1	3,016	LP						
P4	ND	arr 8p23.3p23.2(158,048-5,041,417)x1	4,883	VUS	2	2	_	4 bp microhomology	Inv dup del	seq[GRCh37]+der(8)(p11.2 → p23.2::p23.2 → qter)
		arr 8p23.2p11.21(5,065,495-40,154,240)x3	35,089	P						
P9	ND	arr 11q14.3q25(90,683,052-133,794,488)x3	43,097	P	2	2	IGSF9B	blunt fusion	Inv dup del	seq[GRCh37]+der(11)(11pter → q25::q25 → q14.3)
		arr 11q25(133,795,218-134,938,470)x1	1,143	VUS						
P11	ND	arr 1q43q44(241,711,342-243,902,894)x3	2,192	VUS	4	4	AKT3; CNST; KMO	4bp microhomology; 1 blunt fusion	Inv dup del	seq[GRCh37]+der(1)(pter → q44::q44 → q43::q44)
		arr 1q44(243,908,946-246,761,153)x1	2,852	P						
P5	ND	arr 4p16.3p14(68,345-40,491,786)x3	40,423	P	2	2	_	a 4bp of templated insertion	Products of pericentric inversions	seq[GRCh37]+der(4)(pter → p14::q34.2 → pter)
		arr 4q34.2q35.2(176,481,945-190,957,473)x1	14,476	P						
P8	46,XY,r(18) dn	arr 18p11.32p11.21(136,227-11,032,975)x1	10,897	P	10	8	_	13 bp of templated insertion	Ring chromosome	seq[GRCh37]+mos r(18)(::p11.21 → q23::)/r(18;18) (::p11.21 → q22.1::p11.21 → q22.1::) /r(18)(::p11.2 → q22.1::p11.21 → p11.21::)
		arr 18q22.1q23(61,923,592-78,013,728)x1	16,090	P						
P6	ND	arr 19q13.12q13.2(36,137,595-40,007,825)x3	3,870	LP	6	6	FXYD1; ETV2; CEACAM6	19 bp and 13 bp of non-templated insertions; 1 blunt fusion	Interstitial deletion/duplication/triplication	seq[GRCh37]+der(19)(pter → q13.12::q13.2 → q13.12::q13.2 → 13.2::q13.12 → qter)
		arr 19q13.2(40,011,889-42,290,899)x4	2,279	LP						
		arr 19q13.12(35,630,463-36,134,017)x1	503	LB						
P7	ND	arr 3q27.1q28(184,268,193-190,462,027)x1	6,194	LP	4	7	_	4 bp microhomology; a 4 bp of templated insertion	Interstitial deletion/duplication/triplication	seq[GRCh37]+der(3)(pter → q27.1::q28 → q28::q28 → qter)
		arr 3q28q29(190,469,583-193,995,515)x3	3,526	VUS						
P3	ND	arr 6p25.3p22.3(383,951-17,194,391)x3	16,810	P	4	2	LOXL2	OR cluster; 1 bp microhomology	Complex structural rearrangements	seq[GRCh37]+der(8)(6p25.3 → 6p22.3::8p21.3 → 8p23.1::8p23.1 → 6qter)
		arr 8p23.3p23.1(158,048-6,999,114)x1	6,841	P						
		arr 8p23.1p21.3(12,490,998-23,256,845)x3	10,766	P						
P12	ND	arr 18p11.23(136,227-7,974,486)x3	7,838	P	6	4	DOK6; PTPRM	70 bp of non-templated insertion; 1 blunt fusion	Complex structural rearrangements	seq[GRCh37]+der(18)(pter → q22.2::q22.2 → 21.32::p11.23 → p11.23::pter)
		arr 18q21.32q22.2(58,106,862-67,472,322)x3	9,365	P						
		arr 18q22.2q23(67,474,012-78,013,728)x1	10,539	P						
P14	46,XX,der(9) dn	arr 2q37.2q37.3(236,318,077-242,782,258)x3	6,464	LP	4	4	_	4bp of nontemplated insertion; 5bp microhomology	Complex structural rearrangements	seq[GRCh37]+der(9)(9p13.1 → 9p23.1::9p13.1 → 9q13::2q37.2 → 2q37.3::9q13 → 9qter)
		arr 9p24.3p23(208,454-11,583,419)x1	11,375	P						
		arr 9p23q13(11,583,628-68,323,909)x3	56,740	P						
**(2) CASES WITH NO NEW RESULTS AFTER WGLCS**										
**Patient number**	**Karyotype**	**CMA results (hg19)**	**Length (kb)**	**ACMG classification**	Breakpoints number defined by WGLCS^*^	**Verified by Sanger sequencing**	**Disrupted gene**	**Breakpoint characteristics**	**Structural rearrangements type**	**Final interpretation**
P2	47,XY,+marker	arr 11q23.3q25(116,683,754-134,937,416)x3	18,254	P	2	0	_	AT-rich repeats	+der(22)t(11;22)	+der(22)(22pter → 22q11.21::11q23.3 → 11qter)
		arr 22q11.1q11.21(16,888,899-20,312,661)x3	3,424	P						
P15	47,XY,+marker	arr 11q23.3q25(116,683,754-134,937,416)x3	18,254	P	2	0	_	AT-rich repeats	+der(22)t(11;22)	+der(22)(22pter → 22q11.21::11q23.3 → 11qter)
		arr 22q11.1q11.21(16,888,899-20,312,661)x3	3,424	P						
**(3) CASES WITH STILL UNCLEAR ABERRATION AFTER WGLCS**										
P10	ND	arr 17p11.2(16,591,259-18,629,013)x3	2,037	P	_	_	_	_	_	_
		arr 17p11.2(18,643,290-21,690,654)x4	3,047	P						
P13	46,XY, der(21) dn	arr 21q21.1q22.11(22,782,451-34,401,642)x1	11,619	P	20	8	_	_	_	_
		arr 21q22.3(44,094,699-45,194,949)x1	1,051	VUS						

*CMA, chromosomal microarray; WGLCS, whole-genome low-coverage sequencing; P, pathogenic; LP, likely pathogenic; VUS, variants of uncertain significance; LB, likely benign; ND, not detect*.

**Figure 1:**
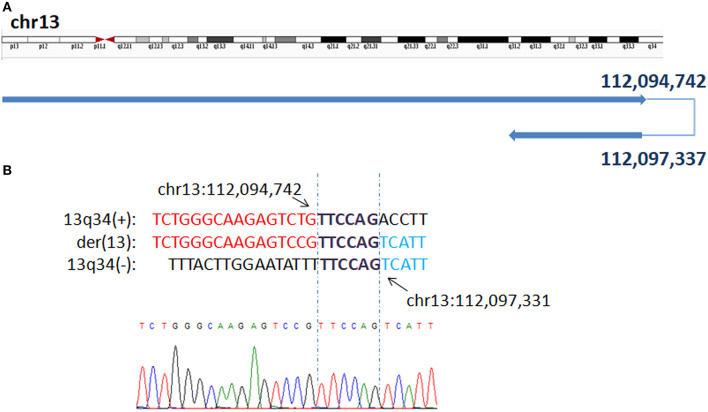
Breakpoints in P1 **(A)** Breakpoints analysis found two breakpoints were at chr13q34:112,094,742 and chr13q34: 112,097,337, which demonstrate inv dup 13q31.2q34 and 13q34qter deletion on der 13 sequences. **(B)** The breakpoints mapped at the base-pair level by Sanger sequencing. Sequences in bold purple represent microhomology. Rearrangement junction sequences (middle line) and matching reference sequences (top and bottom lines) are shown with different colors depending on the involved chromosome region.

**Figure 2:**
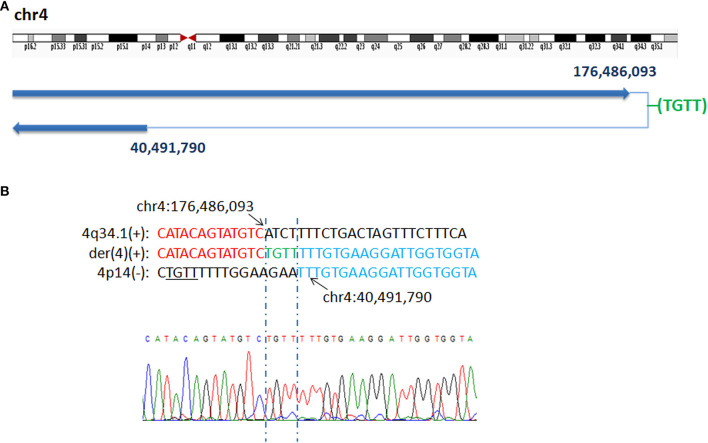
Breakpoints analysis and validation in P5. **(A)** Sanger sequencing of the junction fragments confirmed that the breakpoints on chromosome 4 were located at chr4q34.2: 176,486,093 and chr4p14: 40,491,790. **(B)** The breakpoints mapped at the base-pair level by Sanger sequencing. Rearrangement junction sequences (middle line) and matching reference sequences (top and bottom lines) are shown with different colors depending on the involved chromosome region (4q34.1-red, 4p14-blue). The breakpoint site is indicated in blue line. Der4(+) indicates the junction sequences near the centromere. Green letter, insertion. Sequence with black underline indicate the potential origin of the insertion at junction.

**Figure 3:**
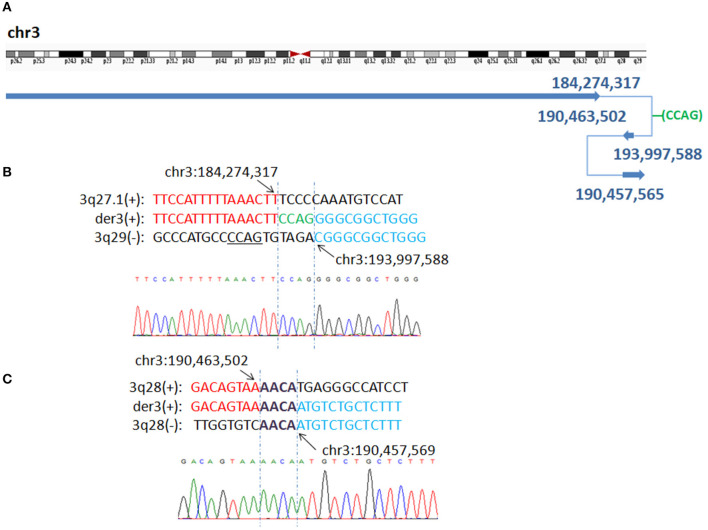
P7 with interstitial deletion/duplication rearrangements. **(A)** Breakpoints analysis and validation found four breakpoints in patient 7: chr3q27.1: 184,274,317, 3q29:193,997,588, 3q28:190,463,502 and 3q28:190,457,565. There is a 4 bp insertion of unknown origin between chr3q27.1: 184,274,317 and 3q29:193,997,588 fusion breakpoint. **(B)** The breakpoint junction 1 mapped at the base-pair level by Sanger sequencing. Rearrangement junction sequences (middle line) and matching reference sequences (top and bottom lines) are shown with different colors depending on the involved chromosome region (3q27.1-red, 3q29-blue). Green letter, insertion. Sequnece with black underline indicate the potential origin if the insertion at junction 1. **(C)** Rearrangement junction 2 sequence (middle line) and matching reference sequences (top and bottom lines) are shown with different colors depending on the involved chromosome region (3q28 tel-red, 3q28 cen-blue). The breakpoint site is indicated in blue line. Der3(+) indicates the junction sequences near the centromere. Sequences in bold purple represent microhomology.

**Figure 4:**
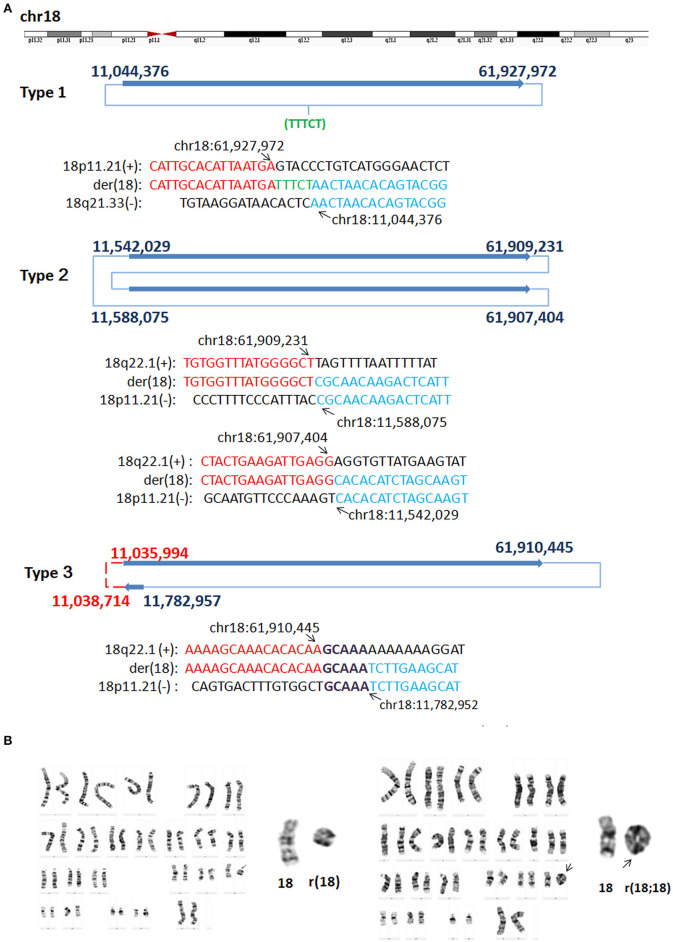
G-band Karyotypes and breakpoints in P8. **(A)** Breakpoint analysis and validation found three types of rings generated from different breakpoints. Type one: the detailed breakpoint sites were validated to be at 11,044,376 bp (18p11.21) and at 61,927,972 bp (18q22.1), respectively by Sanger sequencing, and an 5 bp insertion was found. Green letter, insertion. Type two: the detailed breakpoint sites were validated to be 11,542,029(18p11.21), 61,909,231(18q22.1), 11,588,075 (18p11.21), and 61,907,404 (18q22.1), respectively. Type three: Two breakpoints were validated to be 61,910,445 bp (18q22.1) and 11,782,957 (18p11.21), the other two breakpoints including 11,035,994 (18p11.21) and 11,038,714 (18p11.21) could not be validated by Sanger sequencing (marked as red). Sequences in bold purple represent microhomology. **(B)** Karyotyping showed mosaicism on chromosome 18. The ring 18 (left) and ring (18;18) (right) comprised the mosaicism.

**Figure 5:**
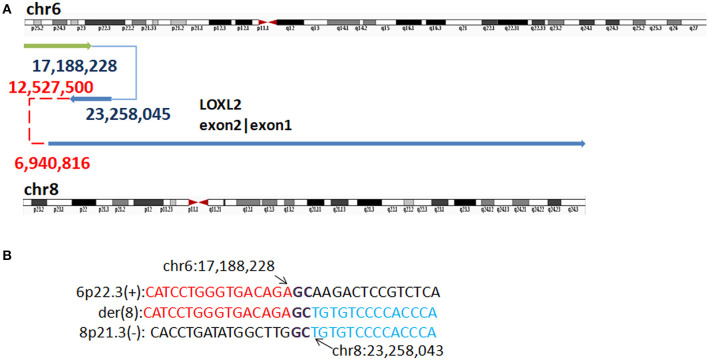
Breakpoints in P3. **(A)** Breakpoints analysis found four fusion breakpoints: 17,188,228 (6p25.3), 23,258,045 (8p21.3), 12,527,500 (8p23.1), and 6,940,816(8p23.1), and in which chr8: 12,527,500 and 6,940,816 were not validated by Sanger sequencing (marked as red). **(B)** The breakpoints mapped at the base-pair level by Sanger sequencing. Rearrangement junction sequences (middle line) and matching reference sequences (top and bottom lines) are shown with different colors depending on the involved chromosome region. Sequences in bold purple represent microhomology.

* *breakpoint junctions refined by WGLCS were listed in Table S2 and Figure 1-5, and supple images*.

### Chromosomal Microarray Analysis

Fourteen patients were analyzed by CytoScan™ 750 k or HD (Affymetrix Inc., Santa Clara, California, USA). One patient was analyzed using the Agilent 4 × 44K (Santa Clara, California, USA). Genomic DNA was extracted from peripheral blood using QIAamp DNA Blood Midi Kit (Qiagen, CA, USA). The chromosomal microarray experiment was performed using protocols provided by the manufacturer. Affymetrix® Chromosome Analysis Suite 1.2.2 (Affymetrix Inc.) and Genomic Workbench software (Agilent, Inc) were used to detect and analyze the chromosomal CNVs identified in the patients. The chromosome positions are shown according to GRCh37 (hg19).

### Whole-Genome Low-Coverage Sequencing and Bioinformatics Analysis

The procedures for sample preparation, sequencing, and data analysis were performed as previously described by Dong et al. (2014). In short, the genomic DNA was fragmented into ~4 kb in size by ultrasound (Covaris, Woburn, MA USA). Each fragment was circlized with a biotin labeled ring adapter (sequence: CTGTCTCTTATACACATCTAGATGTGTATAAGAGACAG) by T4 ligase (New England Biolabs, Ipswich, MA USA). The ring was further broken into ~250 bp fragments by Covaris. Streptavidin-coupled Dynabeads (Invitrogen, Waltham, MA USA) was applied to isolate fragments containing the ring adapter. The isolated fragments were consequently sequenced by Illumina HiSeq2000 (IIlumina, San Diego, CA USA), and 150 bp paired end reads were generated. The raw data was first trimmed by fastx-toolkit (0.0.14) to remove adaptor. The trimmed fastq files were aligned to hg19 reference genome by BWA (0.7.12). The bam files were manipulated by picard (1.124) to remove duplicates. CNVkit (0.9.2.dev0) and Pindel (0.2.5b9) were applied to call CNVs and to infer break point junctions of chromosomal rearrangements.

### Verification of Chromosomal Rearrangements by PCR and Sanger Sequencing

Breakpoints were confirmed using PCR amplification and Sanger sequencing of junction fragments. PCR primer sequences and protocols are available upon request. Amplified fragments were sequenced using a 96-capillary 3730xl system (Applied Biosystems, Waltham, MA USA).

## Results

In 15 ID/DD patients, CMA found and reported 34 CNVs. Each patient had an average of 2–3 pathogenic CNVs involving 1–2 chromosomes (Table 1). The pathogenicity of all described CNVs was determined by using ACMG guidelines (Riggs et al., 2020). WGLCS discovered all the pathogenic CNVs found by CMA (Table S1) and identified 70 breakpoints in these patients, of which 47 were finely mapped at the nucleotide level and were confirmed by subsequent PCR amplification and Sanger sequencing of junction fragments (Table S2). Gene disruptions were detected in 15 out of 47 breakpoints involving 10 genes. Among them, 14 breakpoints located in introns and one in exon (Table 1). For the 22 breakpoints unconfirmed by Sanger sequencing, 12 breakpoints were from one patient with complex chromosomal rearrangements. For these unconfirmed breakpoints, 20 were mapped to contig gaps, which could not be aligned and mapped. The other two located in intronic regions. We amplify this junction but failed to get the exact sequences due to a strand of T in the sequences.

In 15 patients, we divided the results into three categories: (1) 11 patients (P1, P4, P9, P11, P5, P8, P6, P7, P3, P12, and P14), WGLCS revealed new breakpoints or finely mapped the genes disrupted by chromosomal rearrangements or microhomology and/or short insertion at the breakpoint junctions; (2) No additional information (breakpoints or structural changes) in two patients (P2 and P15) was revealed by WGLCS; (3) Structural rearrangements in the other two patients (P10, P13) were still unclear after WGLCS was performed (Table 1). The structural rearrangement identified in the 13 patients with clear results could be divided into the following categories: (1) interstitial inverted duplication with concomitant terminal deletion (inv dup del) (P1, P4, P9, and P11); (2) the product of pericentric inversion (P5); (3) interstitial duplication and/or triplication (P6 and P7); (4) ring chromosome (P8); and (5) +der(22)t(11;22) (P2 and P15); (6) complex structural rearrangements (P3, P12 and P14); (7) undetermined structural rearrangements (P10 and P13).

The results in 15 patients were divided into three categories after WGLCS was performed:

**(1) Patients with new results revealed by WGLCS**

In P1, WGLCS revealed the breakpoints to chr13: 112,094,742 and 112,097,337, which demonstrate inv dup 13q31.2q34 and 13q34qter deletion on der 13 sequences (Figure 1). There is a 2.6-kb disomic region between duplication and deletion. A 6 bp microhomology (TTCCAG) at the breakpoint junction was found.

In P4, the breakpoints can be refined to chr8: 5,062,619 and 5,061,157, which demonstrated inv dup 8p23.2p11.21 and 8p23.2pter deletion on der 8 sequences (Figure S3). There is a 1.5-kb disomic region between duplication and deletion. There is 4 bp microhomology (ATTA) was found at recombinant junctions.

In P9, WGLCS found two fusion breakpoints to chr11q25: 133,787,448 and 133,789,046, which disrupted the *IGSF9B* gene in intron 18. These data demonstrate inv dup 11q14.3q25 and 11q25qter deletion on der 11 chromosome. No repeat or microhomology was found at recombinant junctions (Figure S4).

WGLCS refined four fusion breakpoints in P11, chr1q44:243,902,251, chr1q44:243,905,812, chr1q43:241,709,795, and chr1q44:246,760,357. The results demonstrated inv dup 1q43q44 and partial 1q44 deletion on der 1 chromosome. Breakpoints in 1q44 disrupted the *AKT3* gene in intron 1 and *CNST* gene in intron 2. There is a 4 bp microhomology (TGGA) at first junction and a 3 bp microhomology (TTC) at second junction (Figure S5).

In P5, WGLCS identified two fusion breakpoints on chromosome 4 to chr4q34.2: 176,486,093 and chr4p14: 40,491,790. A 4 bp inserted sequence (TGTT), which possibly originated from a nearby sequence (chr4:40,491,801–40,491,804), at the breakpoint junction were found (Figure 2).

In P6, we found six breakpoints at chr19q13.12:35,632,662, 36,134,620, 36,136,056 and chr19q13.2: 42,279,851, 42,274,891, and 40,015,980. We also found a 19 bp insertion of unknown origin between the fusion breakpoints chr19q13.12: 36,134,620 and chr19q13.2: 42,274,891 and a 13 bp insertion of unknown origin between the fusion breakpoints chr19q13.2: 40,015,980 and chr19q13.12: 36,136,056. The breakpoint at chr19q13.12: 35,632,662 disrupted the *FXYD1* gene (Figure S6).

WGLCS identified four breakpoints in P7, namely, chr3q27.1: 184,274,317, chr3q28: 190,463,502, 190,457,565 and chr3q29: 193,997,588. There was a 4 bp insertion, which possibly originated from a nearby sequence (chr3: 193,997,596-193,997,599), between the fusion breakpoints chr3q27.1: 184,274,317 and chr3q29: 193,997,588. A 4 bp microhomology (AACA) was found at the second breakpoint junction (Figure 3).

In P8, WGLCS analysis suggested three types of rings generated from different breakpoints in chromosome 18 (Figure 4). Type one: the detailed breakpoint sites were validated at 11,044,376 bp (18p11.21) and 61,927,972 bp (18q22.1) with an 18 bp insertion, in which 13 bp possibly originated from a nearby sequence (chr18:61,927,960–61,927,972), was found between fused breakpoints. Type two: the detailed breakpoint sites were validated at 11,542,029(18p11.21), 61,909,231(18q22.1), 11,588,075 (18p11.21), and 61,907,404(18q22.1), respectively. We could infer a karyotype of der(18) r(18;18) (::p11.21 → q22.1::p11.21 → q22.1::). Type three: Two breakpoints were detected and validated to be 61,910,445 bp (18q22.1) and 11,782,957 bp (18p11.21), and a 5 bp microhomology (GCAAA) was found at this breakpoint junction. The other two breakpoints including 11,035,994 bp (18p11.21) and chr18: 11,038,714 (18p11.21) could not be validated by Sanger sequencing.

In P3, WGLCS detected four fused breakpoints: 17,188,228 (6p25.3), 23,258,045 (8p21.3), 12,527,500 (8p23.1), and 6,940,816 (8p23.1), in which chr8: 12,527,500 and 6,940,816 were not validated by Sanger sequencing (Figure 5). Two breakpoints at 8p23.1 were located at olfactory repeat clusters. A 1 bp microhomology (G) between the breakpoint junction of 6p25.3 and 8p21.3 was found.

P12 harbors six fusions: chr18q22.2: 67,471,373 and 67,481,632, chr18q21.32:58,112,699, chr18p11.23:7,902,199, chr18p11.23:7,977,760, and chr18p11.23: 7,840,389. The first junction, chr18q22.2: 67,471,373 and 67,481,632 could not be validated by Sanger sequencing. Breakpoint at 18q22.2 disrupts *DOK6* gene in intron 7. In addition, a 70 bp insertion of unknown origin was found between the fusion breakpoints of chr18p11.23: 7,977,760 and 7,840,389 (Figure S7).

In P14, WGLCS detected four fusion breakpoints, namely chr9p23:11,569,433, chr9p23: 11,576,464, chr9q13: 44,302,981 or 46,744,925 (the two genomic regions are homolog, so it is unable to know the exact location by short reads), and chr2q37.2: 236,328,203, which demonstrated inv dup 9p23p13.1 and 9p23pter deletion with a 2q37.2q37.3 insertion at 9q13 on der9 chromosome (Figure S8). There is a 4 bp insertion (CATT), which possibly originated from a nearby sequence (chr2:236,328,190–236,328,193), between the fused breakpoints chr9q13: 44,302,981 or 46,744,925 and chr2q37.2: 236,328,203. A 5 bp microhomology (GATGG) was found at breakpoint junction of 9p23 (Figure S8).

**(2) Patients with no additional results revealed by WGLCS**

In P2 and P15, WGLCS found two derivative sequences (der 11 and der 22, respectively), and identified a breakpoint near 11q23.3q25 (chr11: 116,683,298) and another breakpoint near 22q11.21 (chr22: 20,326,114) (Figures S1, S2). However, the breakpoints could not be finely mapped because the breakpoint sequences of both chromosomes contained hundreds of base pairs of palindromic AT-rich repeats (PATRRs). Routine G-band chromosome analysis of P15 confirmed an extra marker chromosome with unknown origin, and parental karyotyping analysis showed that the mother of the patient was a balanced carrier of t(11;22)(q23.3;q11.2). These data suggest that the derivate sequences were from a small supernumerary marker chromosome +der(22)t(11;22)(q23.3;q11.21) mat (Figure S2).

**(3) Patients with structural rearrangements still unclear after WGLCS was performed**

In P10, microarray identified an intrachromosomal duplication at 17p11.2 (16,591,259–18,629,013), and partial triplication at 17p11.2 (18,643,290–21,690,654), but WGLCS failed to map the breakpoints located at the N region in hg19.

In P13, we found two interstitial deletions at 21q21.1q22.11 (21,395,672–33,089,005) and 21q22.3 (44,080,000–45,857,506) and two small duplications between these two deletions. WGLCS reads revealed approximately 20 breakpoints, but only 8 breakpoints could be confirmed by Sanger sequencing (Figure S9). Owing to complex rearrangements and the location of some breakpoints in the N region, all the breakpoints could not be finely mapped.

## Discussion

WGLCS managed to detect all the copy number variations found by CMA and enabled the identification of most breakpoint of the structure changes in one experiment. Gene disruptions were detected in 15 out of 48 confirmed breakpoints (Table 1), in which 14 occurring within introns of nine genes and one in exon of *ETV2* genes. Among these 10 genes, *AKT3* gene, disrupted in P11, is associated with Megalencephaly-polymicrogyria-polydactyly-hydrocephalus syndrome 2 (MPPH2) which is characterized by ID, megalencephaly and bilateral perisylvian polymicrogyria. We reanalyzed the phenotype of this patient. She manifested severe ID, microcephaly, growth retardation, atrial septal defect and normal brain MRI results. So far majority of the disease causing *AKT3* variants are missense that does not lead to loss of gene function. It suggests a gain-of-function mechanism of the mutations. The phenotypes of P11 result from the overall effect of the genomic structure changes instead of *AKT3* gene disruption. Other disrupted genes have not been reported to be associated with known syndrome, which might have little phenotypic consequence.

The formation of a CNV depends on the joining of two formerly separated DNA segments, and these breakpoint junctions yield insights into the mechanisms that cause the chromosomal structural change (Carvalho and Lupski, 2016). In most recurrent rearrangements, the breakpoints clustered within long, highly identical, flanking interspersed paralogous repeats, which mostly consist of low-copy repeats (LCRs) (Carvalho and Lupski, 2016). As for the recurrent 11q23;22q11 translocation, the breakpoint sequences of both chromosomes contained hundreds of base pairs of palindromic AT-rich repeats (PATRRs) which were responsible for translocations (Edelmann et al., 2001). These AT-rich repeats resulted in unable to map breakpoint at base-pair level in P2 and P15 by short reads in this study. Another recurrent rearrangement of inv dup del at chromosome 8p found in P3, the formation of this rearrangement at 8p was mediated by non-allelic homologous recombination (NAHR) between LCR sequences made up of the olfactory receptor gene cluster flanking at the disomic region. In many cases, the mothers of individuals with such a rearrangement carry an inversion between the two olfactory receptor gene clusters, which has an individual recurrent risk (Giglio et al., 2001; Giorda et al., 2007; Rowe et al., 2009). In contrast, P4 with inv dup del 8p had only 1.4-kb segment region of disomy between the duplication and the deletion. Repeats were not found at breakpoints, and the breakpoints in this case also differ from those described in the other 8p cases in which olfactory repeat clusters were identified flanking the disomic region. According the sequencing results of breakpoint junction, a 4 bp microhomology was found which suggested another potential replication-based mechanisms such as fork stalling and template switching/microhomology-mediated break induced replication (FoSTeS/MMBIR), which has been proposed to generate non-recurrent complex genomic rearrangements independent of LCRs (Carvalho and Lupski, 2016). Therefore, not all cases of inv dup del 8p will be mediated by NAHR. In contrast, no repeats or microhomology was found in P9 with inv dup del (11q), this junction might be consequence of double-strand breaks and the reassembly of DNA fragments by repair-based non-homologous end-joining (NHEJ) (Kurtas et al., 2019). Another four patients (P1, P11, P12, and P14) were found with rearrangement of inv dup del, the maximal distance between duplication and deletion was ranged from 2 to 10 kb, which is less than any currently described microdeletion or microduplication syndrome mediated by NAHR between LCRs. Furthermore, we did not detect flanking homologous LCRs for any of the regions. In previous FISH study about inv dup del, U-type mechanism was proposed as the most frequent mechanism for the formation of inv dup del with no obvious disomic region between dup and del region (Rowe et al., 2009). In contrast, the high resolution analysis found 4–6 bp microhomology at the breakpoint junctions in three patients (P1, P11, and P14) and two short insertions at junctions in two patients (P12 and P14). These breakpoint junction features might be explained by potential replication-based mechanisms such as FoSTeS/MMBIR. Microhomology at joining point and short template segments (<100 nucleotides) insertion originated from nearby segments (within 300 bp) at junctions, are the hallmarks of DNA replication-based mechanisms (Zhang et al., 2009; Yuan et al., 2015; Carvalho and Lupski, 2016; Grochowski et al., 2018; Kurtas et al., 2019). Similarly, short insertions originated from nearby sequence and/or microhomology were also found in P5, P7, and P8, together with inversion or intrachromosomal duplication/triplicatation or ring in these patient also propose a DNA replicative/repair mechanism underlying formation of complex intrachromosomal rearrangement. It has been suggested that intrachromosomal template switches have the potential to generate different types of complex genomic rearrangements, such as the insertion of short genomic segments at the repair site, large-scale copy number alterations (for example, duplications, triplications, and higher-order amplifications), and inversions (Carvalho and Lupski, 2016). Therefore, DNA replication/repair mechanism has an important role underlying formation of complex genomic rearrangements, which could be formed in a single mutational event during DNA repair.

The samples used for this study were all ID/DD patients, and CMA has been performed as first-tier diagnostic test in ID/DD patients in clinical practice, so only 5 patients (P2, P8, P13, P14, and P15) in this study have karyotype results. Although the rearrangements identified in this study mostly involved large fragments of DNA (which are cytogenetically visible), the structural changes were not all figured out by conventional karyotyping. In this cohort, about half of the patients had CNV patterns consistent with certain underlying rearrangement mechanism. For example, the gain of 11q and 22q in two patients (P2 and P15) are consistent with the der(11)t(11;22) derived from 3:1 segregation of t(11;22) in Emanuel syndrome (Shaikh et al., 1999). In these two cases, WGLCS was unnecessary. Similarly, the CNV patterns in patients 1, 4, 9, 11, 3, 5, and 8 have been suggested inv dup, pericentric inversion and ring chromosome, which seems that WGLCS did not add much new results in these patients. In fact, WGLCS defines breakpoints and their disrupted genes and surrounding sequences in one experiment at base-pair-resolution. It helps us to learn more about the mechanisms of formation of observed structural rearrangements, and phenotypic consequence of disrupted genes in most of patients. As of note, if WGLCS refined the breakpoints in the patients, followed parental carrier test could be determined by Sanger sequencing covering breakpoints. It is simple, cost- and time-saving compared with the karyotype test in clinical practice. As it could detect structural rearrangements at nucleotide level, it might be applied in preimplantation genetic diagnosis (PGD). For parents with balanced chromosomal rearrangement, the recurrent risk of having children with genomic dosage change is high, so in the next pregnancy, prenatal diagnosis or PGD is recommended. During PGD, primers covering the junction fragment can distinguish normal embryos from those with balanced and unbalanced rearrangements. Then, a genome-wide chromosome test could be provided only for the normal embryo pretested by Sanger sequencing. This application enables chromosomal ploidy and rearrangement carrier status screening of the embryos, and selection of rearrangement-free embryos from individuals carrying chromosomal rearrangements. In addition, this optimal sequential test is relatively economical than genome-wide CNV test for all the embryos. The prerequisite of this sequential test is the probands or parents already tested by WGLCS and breakpoints already mapped at the nucleotide level.

There are some NGS-based methods for mapping breakpoints in disease-associated balanced chromosome rearrangements or pathogenic CNVs associated with unbalanced chromosome rearrangements, such as whole genome paired-end sequencing, which enables to accurately detect balanced chromosomal rearrangement-associated breakpoints, but this technique is highly dependent on prior knowledge of the affected G-band region (Chen et al., 2010; Schluth-Bolard et al., 2013). And another approach mate-pair sequencing, which is a powerful tool to identify copy number abnormalities, translocation, inversion, and complex chromosomal rearrangements simultaneously (Vergult et al., 2014). In theory, whole genome sequencing would enable us to detect numerous variations in the genome at base pair resolution. However, owing to the imperfections in the reference genome and the nature of common short reads from regular NGS, deciphering all the variations in a certain genome remains a difficult task, especially for complex rearrangements, as they frequently involve repeat elements and are a sequencing black hole. WGLCS with large insert size (~4 kb) in this study has similar mechanism of mate pair sequencing. It would overcome small repetitions, but for segmental duplicates and large repeat elements and sequencing gaps, this method would fail as well. In this study, there were 22 breakpoints that could not be confirmed by Sanger sequencing, and 20 of these located in the gaps of the genome that could not be aligned and mapped. Therefore, the main reason accounting for breakpoints that could not be finely mapped is gaps in the reference genome. Another reason is mainly due to the fact that breakpoints lie within segmental duplications or common repeats, which reduces the mapability of short reads. Such as well-known 11q23;22q11 translocation, the breakpoint sequences of both chromosomes contained hundreds of base pairs of palindromic AT-rich repeats (PATRRs) which resulted in unable to map breakpoint at base-pair level in this recurrent rearrangement. In another recurrent inv dup del (8p) caused by NAHR mediated by olfactory receptor gene cluster also hamper precise breakpoint mapping. Similarly, another genomic disorders 17p11.2 microdeletion/duplication leading to Smith–Magenis syndrome/Potocki-Lupski syndrome, in which large LCR (~200 k)-mediated NAHR are responsible for the recurrent deletions/duplication. In some non-recurring cases, junctions are located at 17p-PROX region, which is composed of LCRs sequences flanking an ~141 kb stretch of microsatellite DNA sequences possibly responsible for the nonrecurring rearrangements identified in the 17p proximal region (Yuan et al., 2015). As in our P10, the breakpoints were also located in this region. Therefore, due to the highly complex and repetitive nature of the satellite DNA sequences, which hamper precise breakpoint mapping and sequencing because of short read lengths of regular NGS. To elucidate complex structural variations in such regions, long reads (such as Pacific Biosciences/Oxford Nanopore) or haplotype assembly 10X/single tube long fragment read (stLFR) would be indispensable.

In this study, WGLCS could detect CNVs and the underlying complex chromosomal rearrangements at high resolution in one experiment, which help to learn more about the mechanisms of formation of observed genomic rearrangements, phenotypic consequence of disrupted genes. Characterization of chromosome rearrangements in these patients is helpful for evaluating recurrent risk, which carries great clinical significance for the family.

## Data Availability Statement

The datasets generated for this study are available on request to the corresponding author.

## Ethics Statement

The study protocol was approved by the Ethics Committee of Xinhua Hospital, Shanghai Jiaotong University School of Medicine. Written informed consent to participate in this study was provided by the participants' legal guardian/next of kin. Written informed consent was obtained from the individual(s), and minor(s)' legal guardian/next of kin, for the publication of any potentially identifiable images or data included in this article.

## Author Contributions

YS and YY conceived and designed the study. BX drafted the manuscript. XY performed breakpoints verification. BX, YS, LW, and YF participated in the data analysis. XJ and XG participated in clinical data collection. YY supervised the study. All authors reviewed and approved this submission.

## Conflict of Interest

The authors declare that the research was conducted in the absence of any commercial or financial relationships that could be construed as a potential conflict of interest.
