# Supplementary material for: Whole Genome Low-Coverage Sequencing Concurrently Detecting Copy Number Variations and Their Underlying Complex Chromosomal Rearrangements by Systematic Breakpoint Mapping in Intellectual Deficiency/Developmental Delay Patients
Source: Front Genet. 2020 Jul 6;11:616. doi: 10.3389/fgene.2020.00616 (PMC7357533; doi:10.3389/fgene.2020.00616)
Supplement: Supplementary file 1 [file Data_Sheet_1.docx]

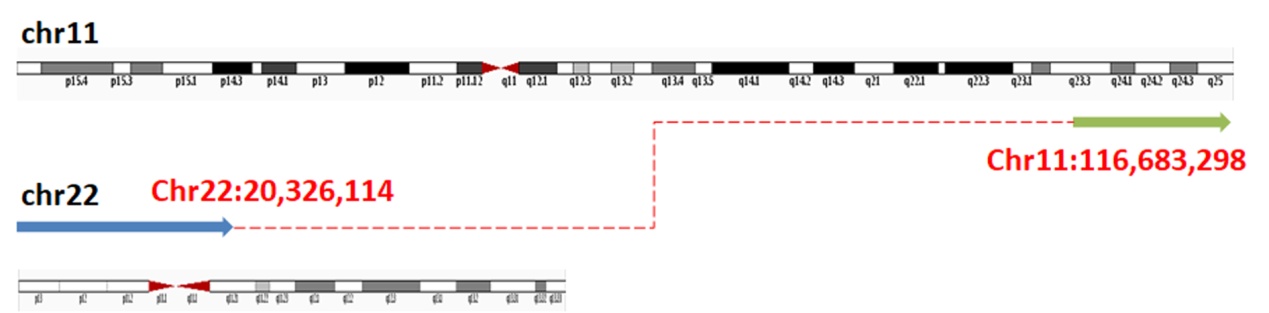


**S1 Fig.** WGLCS found two derivative sequences (der 11 and der 22, respectively) in P2, which further identiﬁed a breakpoint at around chromosome 11q23.3q25 (chr11: 116,683,298) and another breakpoint at around 22q11.21 (chr22: 20,326,114). These two breakpoints could not be finely mapped (marked as red).


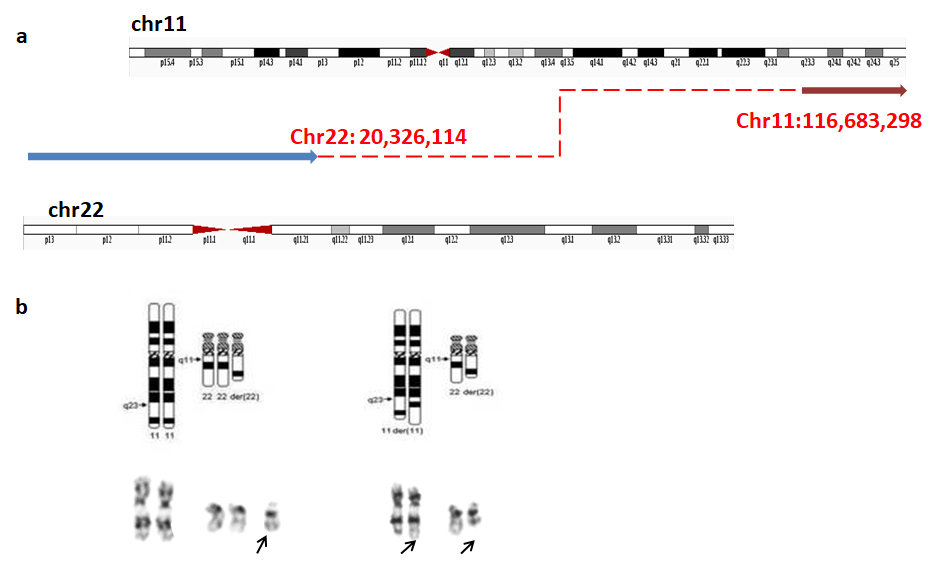


**S2 Fig.** **Breakpoints in P15 and G-banf karyotypes of the patient and his mother. a.** WGLCS found two derivative sequences (der 11 and der 22, respectively) in P15 , which further identiﬁed a breakpoint at around chromosome 11q23.3 (chr11: 116,683,298) and another breakpoint at around 22q11.21 (chr22: 20,326,114)(marked as red). These two breakpoints could not be finely mapped. **b.** Partial karyotyping showed a supernumerary marker chromosome of unknown origin in patient (left, arrow marked), and a reciprocal translocation between chromosome 11q23.3 and 22q11.21 in his mother (right, arrow marked).


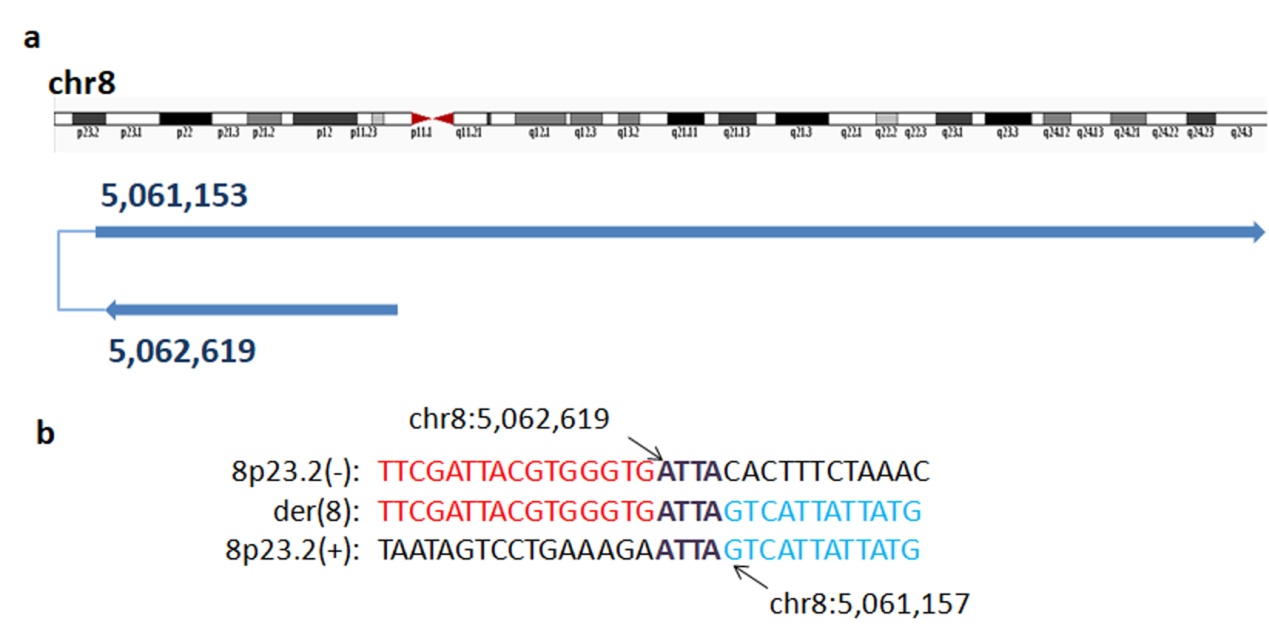


**S3 Fig. Breakpoints in P4. a.** Breakpoints analysis and validation in P4 found two fusion breakpoints: chr8:5,062,619 and chr8: 5,061,153, which demonstrate inv dup 8p23.2p11.21 and 8p23.2pter deletion on der 8 sequences. **b**. The breakpoints mapped at the base-pair level by Sanger sequencing. Rearrangement junction sequences (middle line) and matching reference sequences (top and bottom lines) are shown with different colours depending on the involved chromosome region. Sequences in bold purple represent microhomology.


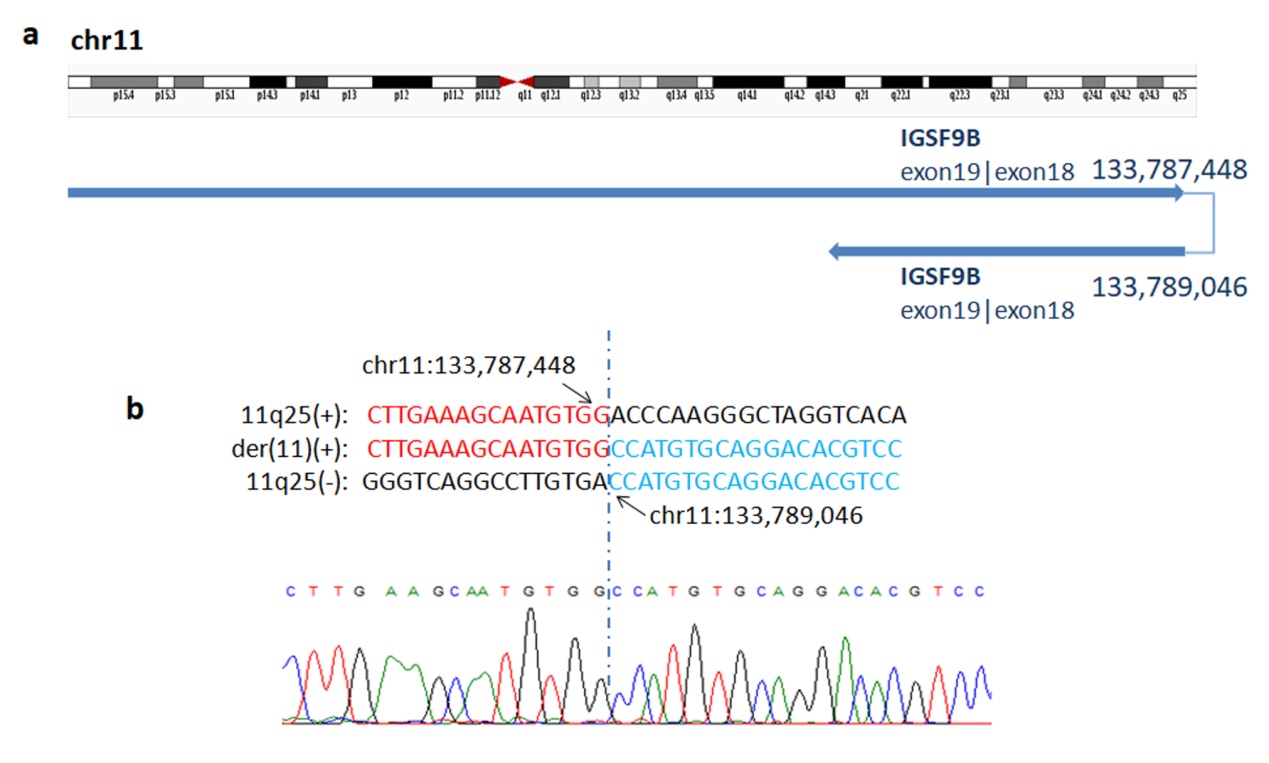


**S4 Fig**. **Breakpoints in P9. a.** Breakpoints analysis and validation found two fusion breakpoints: chr11q25:133,787,448 and chr11q25: 133,789,046 which disrupted IGSF9B gene in intron. These data demonstrate inv dup 11q14.3q25 and 11q25qter deletion on der 11 chromosome. **b.** The breakpoints mapped at the base-pair level by Sanger sequencing. Rearrangement junction sequences (middle line) and matching reference sequences (top and bottom lines) are shown with different colours depending on the involved chromosome region (11q25 near centromere-red, 11q25 near telomere-blue). The breakpoint site is indicated in blue line. Der11(+) indicates the junction sequences near the centromere.


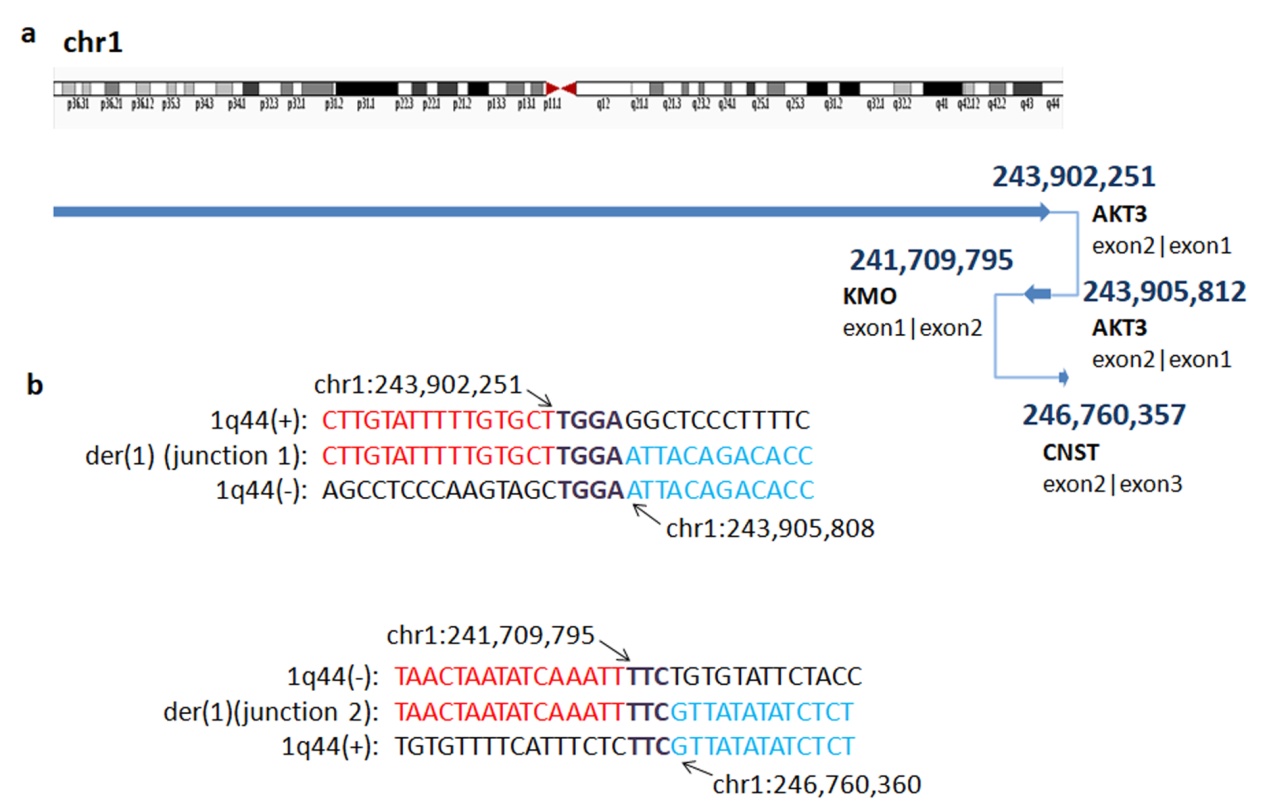


**S5 Fig. Breakpoints in P11. a.** Breakpoints analysis and validation found four fusion breakpoints in P11: chr1q44:243,902,251, chr1q44:243,905,812, chr1q43:241,709,795, and chr1q44:246,760,357, which demonstrated inv dup 1q43q44 and partial 1q44 deletion on der 1 chromosome. Breakpoints in 1q44 disrupts *AKT3* gene in intron1 and *CNST* gene in intron 2, and breakpoint in 1q43 disrupts *KMO* gene in intron 2. **b**. The breakpoints mapped at the base-pair level by Sanger sequencing. Sequences in bold purple represent microhomologies. Rearrangement junction sequences (middle line) and matching reference sequences (top and bottom lines) are shown with different colours depending on the involved chromosome region.


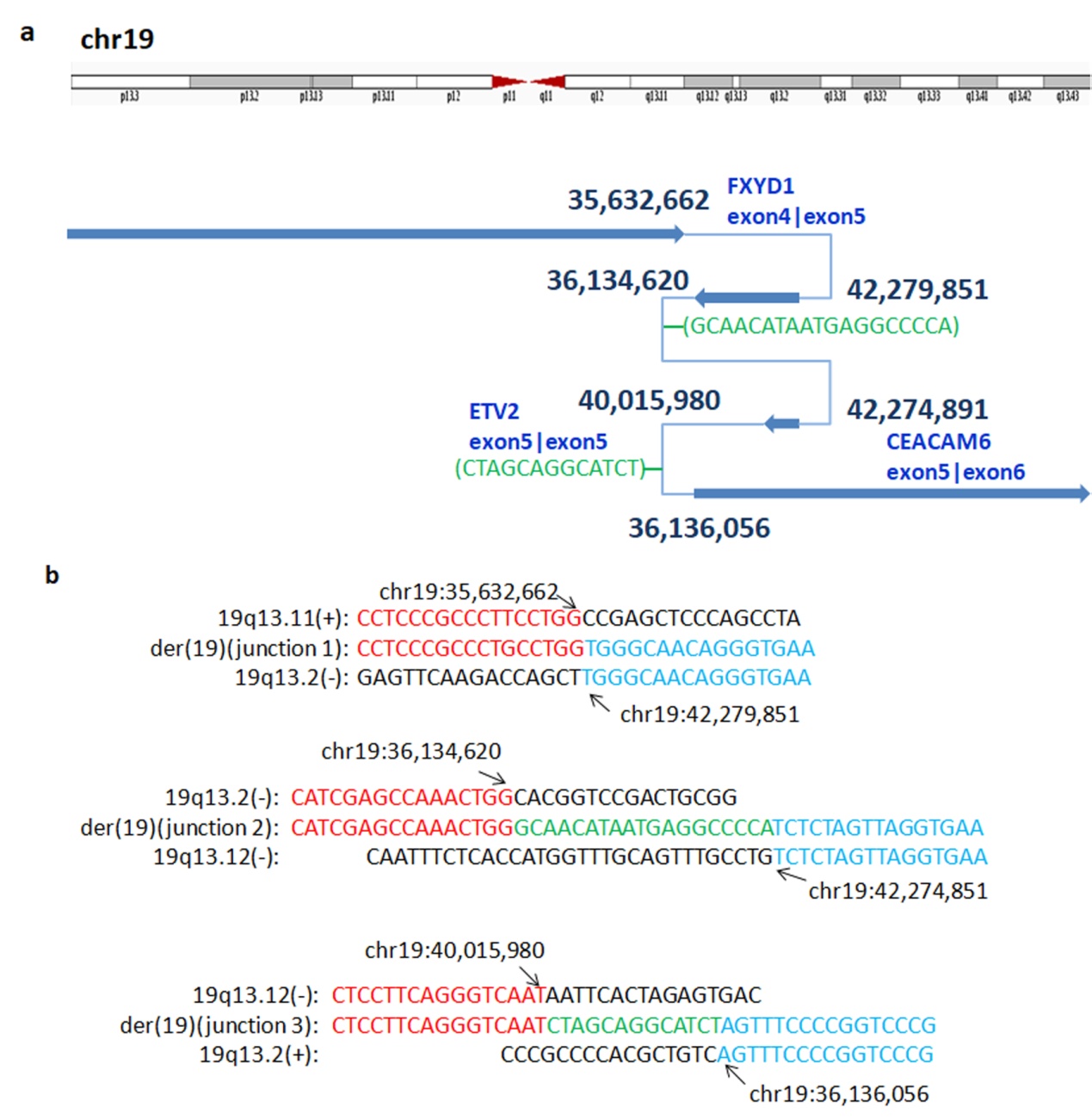


**S6 Fig. Breakpoints in P6. a.** Breakpoints analysis and validation found six breakpoints in patient 6: chr19q13.12: 35,632,662, chr19q13.2: 42,279,851, chr19q13.12:36,134,620, chr19q13.2: 42,274,891, chr19q13.2: 40,015,980, and chr19q13.12: 36,136,056. A 19 bp insertion of unknown origin was found between the fusion breakpoints chr19q13.12:36,134,620 and chr19q13.2: 42,274,891, and a 13bp insertion of unknown origin was found between the fusion breakpoints chr19q13.2: 40,015,980 and chr19q13.12: 36,136,056. The breakpoint at chr19q13.12:35,632,662 disrupts *FXYD1* gene. Green letter, insertion. **b.** The breakpoints mapped at the base-pair level by Sanger sequencing. Rearrangement junction sequences (middle line) and matching reference sequences (top and bottom lines) are shown with different colours depending on the involved chromosome region. Green letter, insertion.


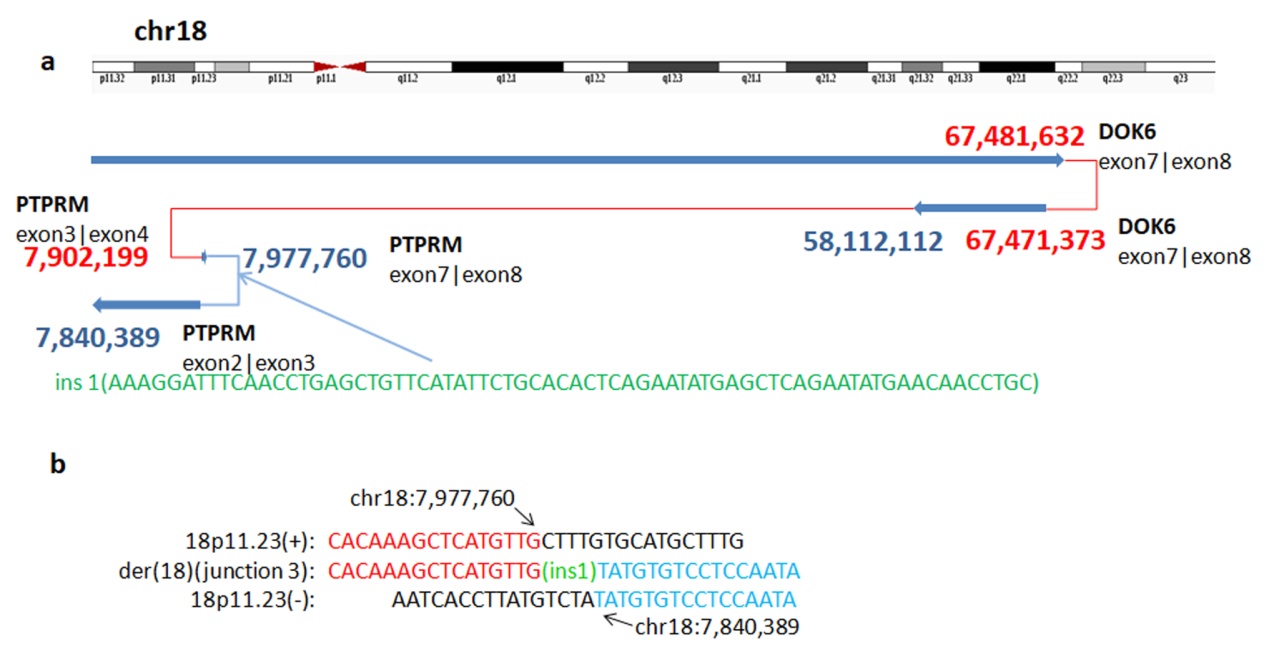


**S7 Fig.** **Breakpoints in P12. a.** Breakpoints analysis found six fusion breakpoints in P12: chr18q22.2:67,471,373 and chr18q22.2:67,481,632, chr18q21.32: 58,112,699, chr18p11.23:7,902,199, chr18p11.23: 7,977,760 and chr18p11.23:7,840,389. In which 67,471,373, 67,481,632, chr18:7,902,199 could not be confirmed by Sanger sequencing (marked as red). A 70 bp insertion of unknown origin between the fusion breakpoints of chr18p11.23: 7,977,760 and chr18p11.23:7,840,389 were found. Breakpoint at 18q22.2 disrupts *DOK6* gene in intron 7. **b.** The two breakpoints mapped at the base-pair level by Sanger sequencing. Rearrangement junction sequences (middle line) and matching reference sequences (top and bottom lines) are shown with different colours depending on the involved chromosome region. Green letters, insertion.


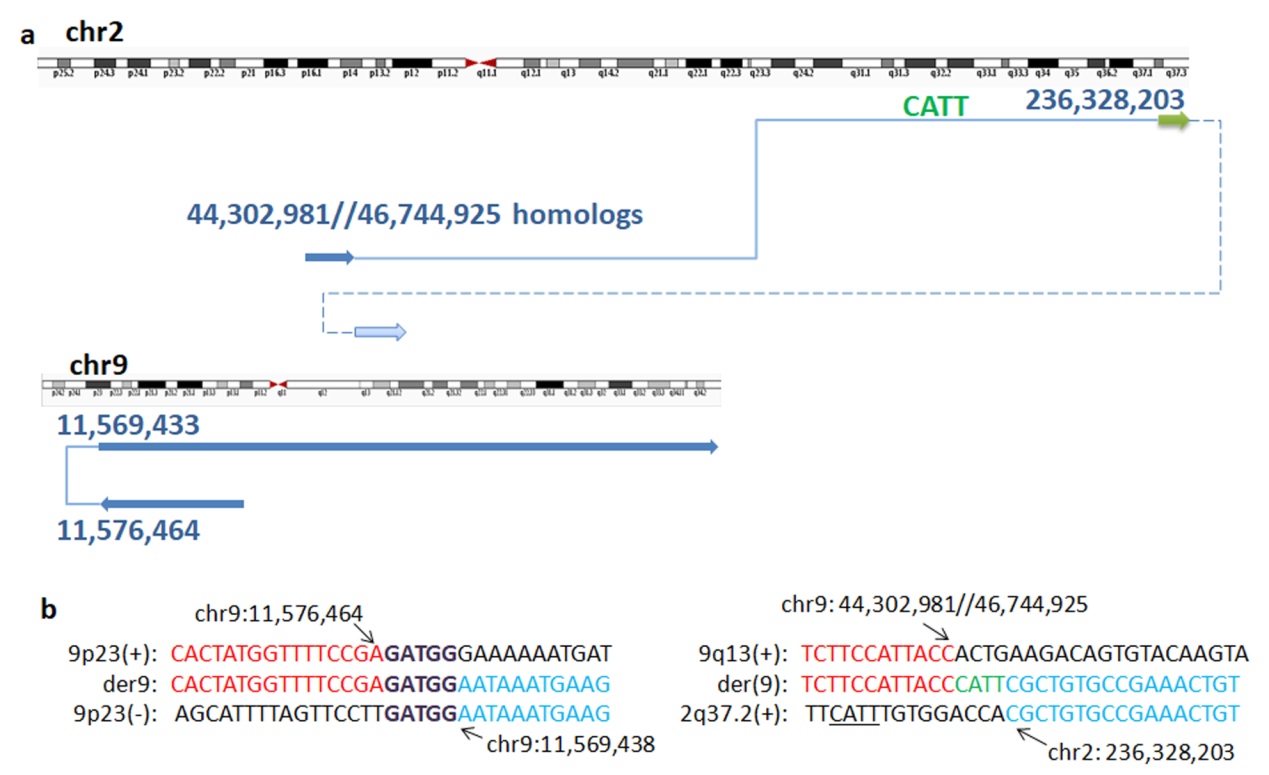


**S8 Fig.** **Breakpoints in P14. a.** Breakpoints analysis and validation found four fusion breakpoints: chr9q13: 44,302,981//46,744,925 and chr2q37.2:236,328,203, chr9p23: 11,569,433 and chr9p23: 11,576,464, which demonstrated inv dup 9p23p13.1 and 9p23pter deletion with a 2q37.2q37.3 insertion at 9q?13 on der9 chromosome. **b**. The breakpoints mapped at the base-pair level by Sanger sequencing. Rearrangement junction sequences (middle line) and matching reference sequences (top and bottom lines) are shown with different colours depending on the involved chromosome region. Green letters, insertion. Sequence with black underline indicate the potential origin of the insertion at junction.


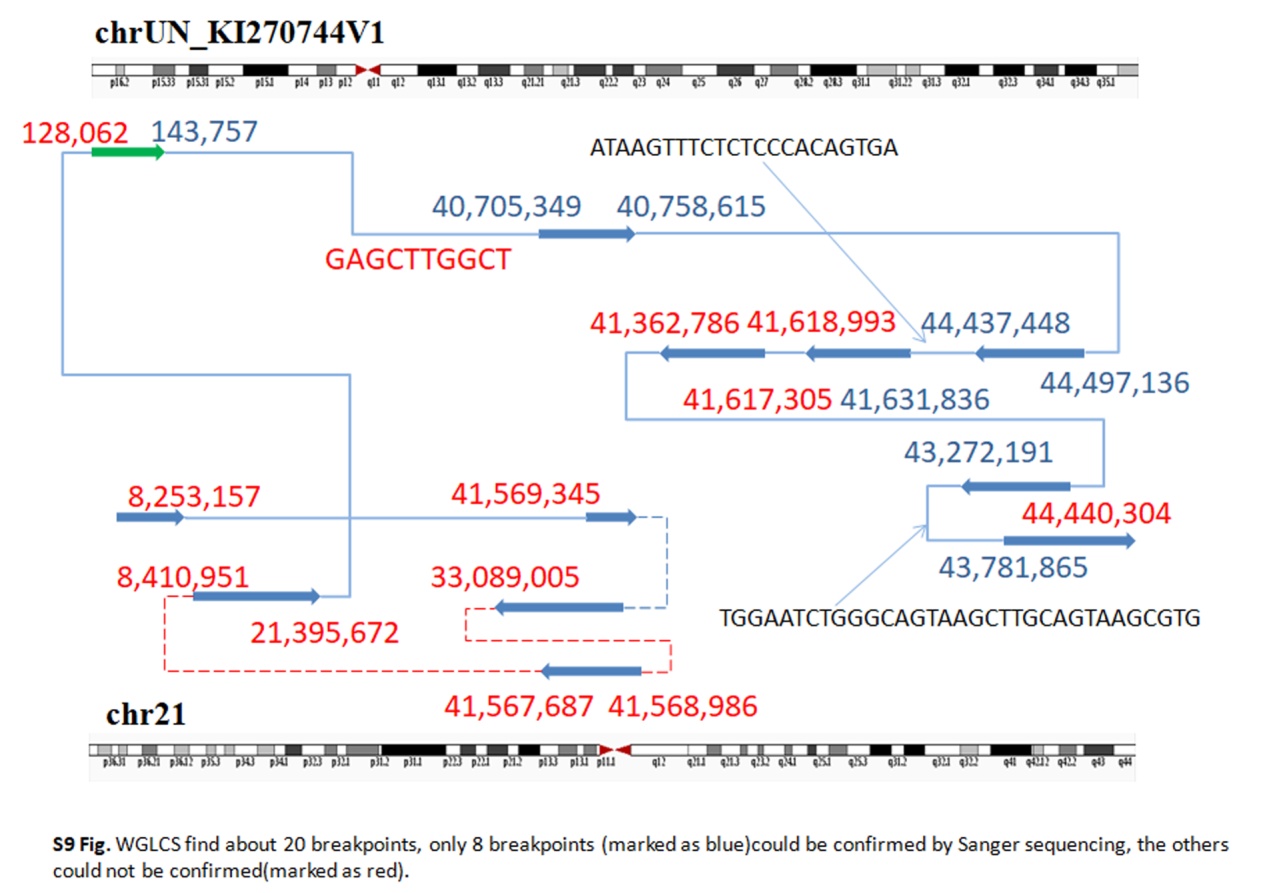


**S9 Fig. Breakpoints in P13.** WGLCS find about 20 breakpoints, only 8 breakpoints (marked as blue) could be confirmed by Sanger sequencing, the others could not be confirmed(marked as red).
